# Sub-optimal host plants have developmental and thermal fitness costs to the invasive fall armyworm

**DOI:** 10.3389/finsc.2023.1204278

**Published:** 2023-09-29

**Authors:** Macdonald Mubayiwa, Honest Machekano, Frank Chidawanyika, Brighton M. Mvumi, Bame Segaiso, Casper Nyamukondiwa

**Affiliations:** ^1^Department of Biological Sciences and Biotechnology, Botswana International University of Science and Technology, Palapye, Botswana; ^2^Department of Zoology and Entomology, University of Pretoria, Pretoria, South Africa; ^3^Plant Health Department, International Centre of Insect Physiology and Ecology (ICIPE), Nairobi, Kenya; ^4^Department of Zoology and Entomology, University of the Free State, Bloemfontein, South Africa; ^5^Department of Agricultural and Biosystems Engineering, Faculty of Agriculture, Environment and Food Systems, University of Zimbabwe, Harare, Zimbabwe; ^6^Department of Zoology and Entomology, Rhodes University, Makhanda, South Africa

**Keywords:** drought-stressed host plants, fall armyworm development, insect diets, intect-plant interactions, subsistence cropping system, thermal responses

## Abstract

The fall armyworm (FAW) *Spodoptera frugiperda* (J.E. Smith) is a global invasive pest of cereals. Although this pest uses maize and sorghum as its main hosts, it is associated with a wide range of host plants due to its polyphagous nature. Despite the FAW's polyphagy being widely reported in literature, few studies have investigated the effects of the non-preferred conditions or forms (e.g., drought-stressed forms) of this pest’s hosts on its physiological and ecological fitness. Thus, the interactive effects of biotic and abiotic stresses on FAW fitness costs or benefits have not been specifically investigated. We therefore assessed the effects of host plant quality on the developmental rates and thermal tolerance of the FAW. Specifically, we reared FAW neonates on three hosts (maize, cowpeas, and pearl millet) under two treatments per host plant [unstressed (well watered) and stressed (water deprived)] until the adult stage. Larval growth rates and pupal weights were determined. Thermal tolerance traits *viz* critical thermal maxima (CT_max_), critical thermal minima (CT_min_), heat knockdown time (HKDT), chill-coma recovery time (CCRT), and supercooling points (SCPs) were measured for the emerging adults from each treatment. The results showed that suboptimal diets significantly prolonged the developmental time of FAW larvae and reduced their growth rates and ultimate body weights, but did not impair their full development. Suboptimal diets (comprising non-cereal plants and drought-stressed cereal plants) increased the number of larval instars to eight compared to six for optimal natural diets (unstressed maize and pearl millet). Apart from direct effects, in all cases, suboptimal diets significantly reduced the heat tolerance of FAWs, but their effect on cold tolerance was recorded only in select cases (e.g., SCP). These results suggest host plant effects on the physical and thermal fitness of FAW, indicating a considerable degree of resilience against multiple stressors. This pest’s resilience can present major drawbacks to its cultural management using suboptimal hosts (in crop rotations or intercrops) through its ability to survive on most host plants despite their water stress condition and gains in thermal fitness. The fate of FAW population persistence under multivariate environmental stresses is therefore not entirely subject to prior environmental host plant history or quality.

## 1 Introduction

Invasive insects face various biophysical stresses throughout the process of naturalisation and invasion (1, 2). Understanding and overcoming these barriers is crucial for the establishment and proliferation of insect species in new environments (3). In the context of tropical and subtropical environments, continuous and repeated bouts of high-temperature stress and seasonal droughts are major barriers limiting the population persistence and establishment of non-native pest insects (2). These factors, coupled with climate change and climate variability, mean that insects are continuously exposed to ever-changing environmental stresses (4–7), including both high- and low-temperature regimes (8, 9) and/or limited or suboptimal food resources (10). These conditions are unfavourable to the invading insects’ physiology, reproductive capacities, and survival (11), which in turn affect insect–host interactions and may have consequences on overall insect fitness and ecology.

Artificial diets (12) and temperature (13, 14) are among the well-studied factors impacting the growth and survival of invasive pests such as the fall armyworm (FAW), *Spodoptera frugiperda* (J.E. Smith) (Lepidoptera: Noctuidae) (14). Previous research on the FAW and fruit flies (*Bactrocera* species) (Diptera: Tephritidae) showed that the natural diets available to insects influence their life-history traits (12, 15, 16). For example, food resources are critical determinants of several insect life-history traits, including progeny number, size, and offspring sex ratios; reproduction timing; weight or size of adults; growth rates; and longevity (17) (**Table S1**). However, the focus of most previous studies has been on artificial diets, such as soybean- or chickpea-based diets (12, 18); only a few have focused on natural hosts such as maize, rice, wheat, star grass, and pinto bean (19, 20). However, in studies that investigated the use of natural host plants, some of the key tropical and subtropical crops such as pearl millet and cowpeas, critical for sub-Saharan African agro-ecosystems, were omitted. Furthermore, previous research did not cover the effects of non-preferred conditions or forms (e.g., drought-stressed forms) of these hosts on the physiological and ecological fitness of associated insect species (21, 22). For example, the interactive effects of biotic (e.g., host quality and type of available food or diet) and abiotic stress (thermal responses due to suboptimal diets) have not been specifically investigated.

In tropical climates, insects are exposed to short-term cold stress and long-term heat stress (8, 9). These stressful environmental conditions may support invasion success or failure thereof (i.e., may shrink or expand the ranges for certain insects depending on species-specific responses) (2, 11). Given the high temperatures in the tropics, heat-stress tolerance is critical for the success of invasive species. Moreover, the tropics have also experienced several anomalous cold waves (9) that are likely linked to climate change. Therefore, surviving low temperatures in the tropics has also become a topical issue. These suboptimal conditions affect the insects not only directly but also indirectly through their host plants (bottom-up effects) and insect–host interactions (23). Drought-stressed diets may influence the direct survival of invasive insects due to their effects on nutritional content and body size (5, 23). However, to the best of our knowledge, no study has investigated the indirect effects of these suboptimal diets (drought-stressed or non-host plants) on the thermal responses of insects (24). Although individual insects with larger body sizes are expected to have higher levels of thermal tolerance than smaller-bodied ones (25), it is generally unknown how individual thermal traits are influenced by suboptimal diets.

The current study used the FAW as a test insect pest. The FAW is a serious pest of several crops, particularly maize and other cereals, and is native to the Americas, but in the last 6 years it has spread to divergent geographic spaces, including most of Africa and parts of the Middle East, Asia, and Australia (26). It is highly polyphagous and an opportunistic feeder, and reports suggest that it has > 350 host plants, including Poaceae, Asteraceae, and Fabaceae (27).

Reports show that 80% to 85% of cowpea is produced in Africa (28–30), often as an intercrop with maize or cereal crops in rural or traditional farming systems (31; reviewed in 32). The FAW is known to attack all growth stages of cowpea (33). A recent study conducted in Botswana by Makale et al. (34) showed that 5% of farmers reported FAW infestation on cowpea, which is comparable to the 9% recorded on sorghum in the same study. Various other studies (35–37) have reported that cowpea is one of the main crops attacked by the FAW in tropical farming areas. In addition to cowpea, the FAW has been reported to feed on a range of related legumes in the family Fabaceae, to which cowpea belongs. For example, 11.3% of the 186 FAW hosts reported by Casmuz et al. (38) belonged to the family Fabaceae. Similarly, 9.9% of the 353 host plants reported by Montezano et al. (27) belonged to the family Fabaceae. These reports provide evidence suggesting that cowpea is one of the key host plants that support the survival of the FAW in African farming systems.

**Figure 1:**
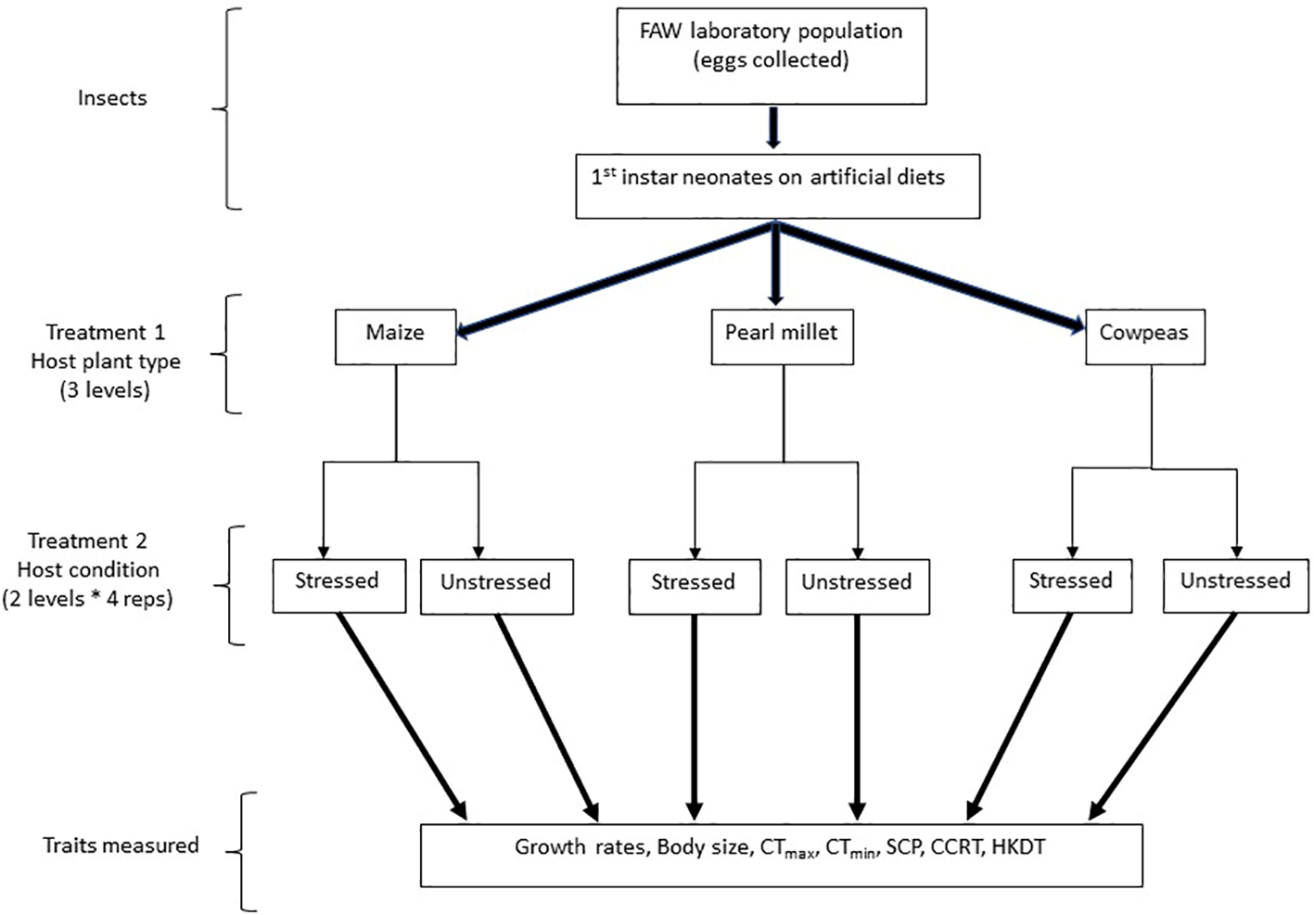
Experimental treatments and traits measured.

Previous studies have shown that exposure to continuous spells of high temperatures and fasting have significant thermal fitness costs on the FAW (39). Nevertheless, most studies to date have dwelt on the effects of artificial diets (12), with a few exceptions (20, 22). Smallholder farmers in sub-Saharan Africa often implement mixed or intercropping systems to optimise land use (40).Therefore, considering how the FAW responds to these context-specific tropical mixed cropping systems is important for tailoring management initiatives. In the current study, we investigated the effects of natural optimal and suboptimal host plants/diets on the ecological and physiological fitness of the FAW. Specifically, we tested the effects of feeding the FAW different host plants (three types: maize, cowpea, and pearl millet), which were either drought stressed or unstressed (**Figure 1**), on the physiological fitness (growth rates and individual size) and thermal stress tolerance of the pest species. Our objective was to assess the life-history traits and thermal fitness traits of FAWs reared on the different host plants (preferred and non-preferred) and different host plant conditions (unstressed and drought stressed). We hypothesised that (1) individuals fed on suboptimal natural diets during larval development may come at ecological and physiological costs to the FAW and subsequent generations; and (2) these fitness costs may be exacerbated when the hosts are drought stressed, which is consistent with the findings of a study conducted by Murua and Virla (41).

## 2 Materials and methods

### 2.1 Experimental insects

A FAW laboratory population of approximately 400 individuals was initiated from specimens collected from smallholder farmers’ fields in Serowe (22°26′00.3''S, 026°49′05.6''E), central Botswana, in January 2022, and was reared for five successive generations in the laboratory. The population was crossed with a wild population (~100 individuals) in May 2022 collected from the same localities to improve their vigour and to avert genetic erosion. This population was then reared for five generations at a temperature of 28°C ± 2°C in the laboratory on an artificial diet containing common bean powder and sorghum leaf powder, as described by Tefera (42). The diet was placed inside 50-mL plastic vials covered with perforated lids for aeration. Approximately 25 first-instar sixth-generation neonates that had hatched on artificial diets (post-crossing with the wild population) were introduced to treatments of different potted host plants, each replicated four times. Treatment host plants were placed in large Mad Hornet cages (Mad Hornet Entomological Supplies, Horne Technologies, Pretoria, South Africa) (142 cm × 58 cm × 58 cm) (see details on this in Section 2.2). Larval growth rates were assessed, and thermal fitness responses were measured for the adults that subsequently eclosed from the pupa originating from the larvae fed on these treatment plants (**Figure 1**).

### 2.2 Host plants

Three host plant types, namely maize (variety SC 602, SeedCo, Gaborone, Botswana), cowpea [variety Tswana Cowpeas, Crosscorn (Pty) Ltd, Gaborone, Botswana], and pearl millet (variety Okashana, SeedCo, Harare, Zimbabwe), were planted in 160 polythene pockets per crop and were watered once every 2 days until 3 weeks after emergence. For each of the three host plants, there were two treatments: (i) “unstressed” and (ii) “drought stressed” (see **Figure 1**). The “unstressed” plants were continually watered once every 2 days to maintain their fresh, unstressed state until the end of the experiment (these were the control plants), whereas the “drought-stressed’ plants were water stressed by watering them only once every 7–10 days (drought-stressed treatment). Each of these two treatments was replicated four times for each host plant (see **Figure 1**). For both groups of plants, basal fertiliser application and all other conditions were kept the same based on farmers’ conventional methods. Following treatment (drought-stressed versus unstressed hosts), these plants were placed inside Mad Hornet breeding cages in a field for experimentation under ambient field conditions. The choice of crops used was based on the preference of mixed farming systems by many farmers in sub-Saharan Africa (as described in 43–45).

### 2.3 Experimental design and data collection

The three types of host plants (maize, cowpea, and pearl millet) and their two types of host condition (drought stressed and unstressed) were used in the experiment (**Figure 1**). Therefore, for host effects on body size and growth rates, the experiment was laid out in a three × two (three host plants × two water stress types) factorial design in a completely randomised design layout, each with four replications (cages). Each cage contained approximately 20 plants. For each replicate, 25 first-instar FAW larvae that had hatched on an artificial diet (42) were introduced to each treatment replicate (in the previously described Mad Hornet cages containing the experimental plants) until the adult stage, and 20 were targeted for data collection). To keep other insects out and contain the experimental individuals, the experiment was conducted in a netshade erected in an open field at the Botswana International University of Science and Technology between November 2022 and January 2023 (austral summer).

Data were collected on ecological traits, including larval weights at different larval instar stages, using head capsule width and ranges as proxies for instar stage following Dyar’s rule (46). Because of their small size, the first and second instars were weighed in groups of ~10 individuals using an ADAM scale (Adam Equipment™ PGW 453e, 450g × 0.001g) to obtain the average weight of individuals. Similarly, fresh pupal weights were measured at two days following moulting to pupation. Furthermore, physiological traits that measure adult tolerance to high and low temperatures (thermal tolerance) were measured (47). These were critical thermal maxima (CT_max_), critical thermal minima (CT_min_), heat knockdown time (HKDT), chill-coma recovery time (CCRT), and supercooling points (SCPs). These were measured using standardised protocols as outlined by Nyamukondiwa et al. (48). Heat knockdown time was measured by acutely subjecting adult insects to a temperature of 50°C and recording the time taken for individuals to lose their self-righting ability (48). For CCRT, the adult insects were subjected to a temperature of 0°C for 1 hour before exposing them to optimal temperature conditions (28°C and 60% RH) and recording the time taken for them to recover. Recovery time was measured as the time taken by the insects to regain self-righting position, e.g., standing on legs, or performing other activities such as locomotion, feeding, and related activities (48). The heat coma (50°C) and chill coma (0°C) temperatures were determined based on the preliminary CT_max_ and CT_min_ experiments. For CT_max_, CT_min,_ and SCPs, an ecologically relevant ramping rate of 0.25°C per minute was chosen, starting from the optimal temperature (28°C) (49) until critical thermal limits or SCPs were recorded (48).

### 2.4 Data analyses

All data were tested for compliance with normality and variance assumptions before analyses using Shapiro–Wilk and Hartley–Bartlett tests, respectively. We tested the homogeneity of our cages (replicates) and the data showed that the replicate cage had no significant effect on all traits (*p* > 0.05), including all interaction effects (*p* > 0.05). Therefore, replicate cage was excluded from further analyses. Data on larval and pupal weights, CT_max_, and CT_min_ met the assumptions of ANOVA for homogeneity of variances and were thus subjected to analysis of variance in Statistica 14.0 (Statsoft Inc., Tulsa, Oklahoma, USA) and means were separated using Tukey’s HSD test. Data on CCRT, HKDT, and SCPs did not meet the assumptions of variance analysis, and hence were analysed using the non-parametric Kruskal–Wallis test. There were no significant differences in CCRT, HKDT, and SCPs between stressed and unstressed plants, and hence the data were pooled in the non-parametric analyses described above at the host plant types. The non-compliant data were log_10_ (**x**)-transformed (where **x** was the original recorded value), before being subjected to a two-way analysis of variance to assess interactive effects. To investigate if relationships existed between pupal weight and thermal fitness traits (CT_max_, CT_min_, HKDT, CCRT, and SCPs), a linear regression analysis was performed in Statistica 14.0.

## 3 Results

### 3.1 Host effects on body size and growth rates

Host plant and stress treatment interactions had significant effects on daily larval weight gain (*p* = 0.014; F_5, 54_ = 4.62), but they had no significant effects on larval weight at the sixth instar stage and pupal weight (**Table S2**). There were significant differences in the growth rates of larval FAWs fed on the different host plants and their stress status (drought stressed or unstressed) (*p* < 0.001). The developmental period was significantly prolonged for larvae fed on cowpea and all stressed host plants (19 days to 25 days, from the sixth instar to the eighth instar) relative to maize and healthier plants (**Table 1**). Larval FAWs fed on unstressed pearl millet and unstressed maize took significantly less time to complete their life cycles (19 and 17 days, respectively, corresponding to six instars).

**Table 1:** Summary table showing FAW larval weights (mean ± SEM) recorded over time following feeding on different natural diets (*n* = 10).

Crop/treatment	Larval weight (g)					
	First instar	Second instar	Third instar	Fourth instar	Sixth instar	Eighth instar
Cowpeas/unstressed	0.01 ± 0.00	0.04 ± 0.01a	0.11 ± 0.00b	0.29 ± 0.04b	0.59 ± 0.01bc	0.62 ± 0.02b
Cowpeas/stressed	0.01 ± 0.00	0.04 ± 0.01a	0.07 ± 0.00ab	0.16 ± 0.02a	0.40 ± 0.02a	0.43 ± 0.01a
Maize/unstressed	0.00 ± 0.00	0.24 ± 0.02b	0.29 ± 0.03c	0.64 ± 0.03d	0.82 ± 0.03d	–
Maize/stressed	0.00 ± 0.00	0.02 ± 0.00a	0.40 ± 0.02d	0.50 ± 0.04c	0.64 ± 0.05bc	0.67 ± 0.05b
Pearl millet/unstressed	0.00 ± 0.00	0.03 ± 0.01a	0.07 ± 0.01ab	0.30 ± 0.02b	0.68 ± 0.06c	–
Pearl millet/stressed	0.00 ± 0.00	0.02 ± 0.00a	0.05 ± 0.00a	0.26 ± 9.04ab	0.54 ± 0.03b	0.59 ± 0.02b
*p*-value	0.193	<.001	<.001	<.001	<.001	0.001
F_5, 53_	1.62	46.74	88.57	25.43	13	10.99

Larval instar stage was determined using head capsule width and ranges as proxies for instar stage following Dyar’s rule (46).

Fall armyworm larvae raised on stressed cowpeas weighed significantly less (0.43 g ± 0.09 g; *p* < 0.001) than those raised on healthier plants. This was followed by stressed pearl millet and unstressed cowpeas, whereas no significant differences were observed between unstressed pearl millet and unstressed maize plants (**Table 1**). In addition, FAWs raised on cowpea hosts recorded the lowest larval weights of the treatments. However, individuals raised on stressed cowpeas recorded significantly lower pupal weights (0.12 g ± 0.01 g) than those raised on unstressed cowpeas (0.14 g ± 0.01 g). The FAW pupal weights on unstressed pearl millet (0.21 g ± 0.01 g) were similar to those from the maize plants (0.20 g ± 0.01 g and 0.23 g ± 0.01 g for stressed and unstressed plants, respectively), whereas those from stressed pearl millet were significantly lower (0.17 g ± 0.01 g) (**Table 1**).

**Table 1**. Mean FAW larval weights (± SEM) recorded over time following feeding on different natural diets (*n* = 10). Larval instar stage was determined using head capsule width and ranges as proxies for instar stage following Dyar’s rule (46).

**Figure 2:**
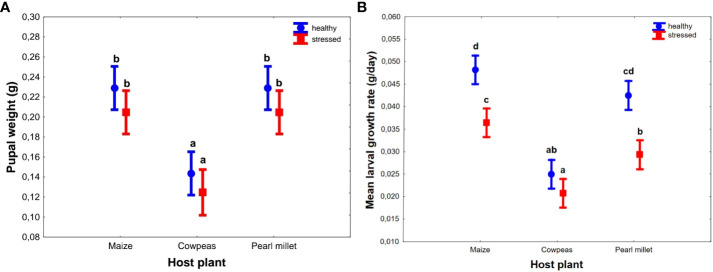
Effects of host plant (three types) and their conditions (two types) on *Spodoptera frugiperda* pupal weight **(A)** and larval growth rates **(B)**. Points represents mean ± SEM. Bars with the same letters were not statistically different from each other across all hosts using Tukey’s HSD test (95% CI).

Host plant had a significant effect on the pupal weight of the FAWs (F_(2, 53)_ = 6.372, *p* < 0.001 (**Figure 2A**). All cereal-based host plants (maize and pearl millet) had significantly higher pupal weights than cowpeas [F_(1, 52)_ = 37.732; *p* < 0.001, **Figure 2A**] regardless of the condition (unstressed or drought stressed) that were not statistically different from each other. However, when the data were pooled, drought stress significantly reduced pupal weight (F_(1, 53)_ = 6.372, *p* = 0.015) regardless of type of host plant (**Figure S1**).

Similarly, cereal host plants showed significantly higher larval growth rates (F_(2, 54)_ = 77.839, *p* < 0.001; **Figure 2B**), with a mean of 0.04 g/day ± 0.002 g/day for both maize and pearl millet, than cowpeas, which was consistent with larval weight results (**Figure 2B**). Drought-stressed plants showed significantly lower larval growth rates, with a mean of 0.029 g/day ± 0.001 g/day, compared to 0.04 g/day ± 0.002 g/day for unstressed plants (F_(2, 54)_ = 56.342, *p* < 0.001), in all cereal host plants but not in cowpeas (*p* = 0.132) (**Figure 2B**).

### 3.2 Effect of host plant and drought stress on heat tolerance

**Figure 3:**
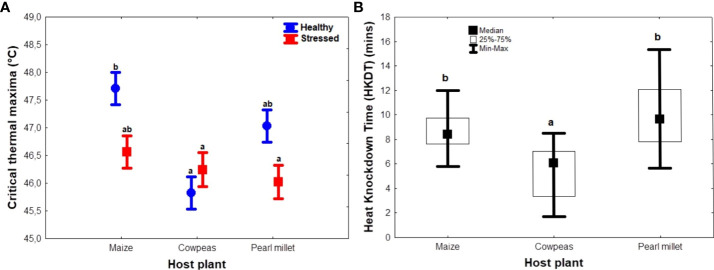
Summary figures showing the effects of natural diet (host plant) and treatment on *Spodoptera frugiperda* heat tolerance **(A)** critical thermal maxima (CT_max_) (across host and stress types), and **(B)** heat knockdown time (HKDT) (across hosts). The circles and boxes in **(A)** represent means for unstressed and stressed plants, respectively, and the error bar represents the standard error of the means. In the boxplots **(B)**, the horizontal bars display the median, the box gives the interquartile ranges, and the whiskers show the largest and smallest values up to 1.5 ×  interquartile range. Stressed and unstressed plant results were pooled for HKDT results since they showed no statistically significant differences. Bars with the same letters are not statistically different from each other at 95% CI (Tukey’s HSD test).

Host plant and stress condition of the plants had significant interactive effects on CT_max_ (F_2, 54_ = 4.51; *p* < 0.05) and HKDT (F_2, 54_ = 27.44; p < 0.001) (**Table 2**). Unstressed maize and pearl millet recorded the highest mean CT_max_ (47.7°C and 47.0°C, respectively), whereas unstressed cowpeas and stressed pearl millet recorded the lowest CT_max_ (45.8°C and 46.0°C, respectively) (**Figure 3A**). On the other hand, unstressed cowpeas recorded the lowest HKDT (3. 37 minutes), whereas unstressed pearl millet recorded the longest HKDT (11. 80 minutes) (**Table S3**) (**Figure 3B**). Overall, FAW larvae raised on unstressed plants and on cereal hosts (maize and pearl millet) generally had higher heat tolerance, measured as CT_max_ and HKDT, than those raised on cowpeas. Thus, host plant type significantly influenced CT_max_ (F_2, 52_ = 7.07, *p* = <0.001), with maize having the highest CT_max_, whereas drought stress significantly reduced the same trait (F_1, 52_ = 5.7, *p* = 0.02), except in cowpeas, in which both unstressed and drought-stressed hosts yielded similar CT_max_ results (**Figure 3A**). FAWs raised on unstressed and stressed cowpeas and stressed pearl millet had the lowest mean CT_max_. Data for the interaction between host plants and their stress conditions on HKDT did not show any significant differences, and thus were pooled for analyses. Individuals raised on cereal host plants (maize and pearl millet) had a significantly higher heat tolerance (longer HKDT) (Kruskal–Wallis test H_(2, N = 55)_ = 22.864, *p* < 0.05; **Figure 3B**) than those reared on cowpeas, which was consistent with larval and pupal weight results. However, drought stress had no significant effect on HKDT (H_(1, N= 55)_ = 0.954, *p* = 0.33) (**Figure 3B**).

**Table 2:** Host plant and stress condition interaction on thermal fitness parameters of FAW adults (mean ± SEM) (n=10).

Dependent variable	Interactive effect	DF	F	*p*-value
Larval growth rate	Host plant × stress types	2, 54	4.618	0.0141
Pupal weight	Host plant × stress types	2, 53	0.038	0.9624
CT_max_	Host plant × stress types	2, 52	4.300	0.0184
CT_min_	Host plant × stress types	2, 53	1.189	0.3124
HKDT	Host plant × stress types	2, 49	24. 079	0.0000
CCRT	Host plant × stress types	2, 48	7. 446	0.0015
SCP	Host plant × stress types	2, 54	11.976	0.0000

### 3.3 Effect of host plant and drought stress on cold tolerance

**Figure 4:**
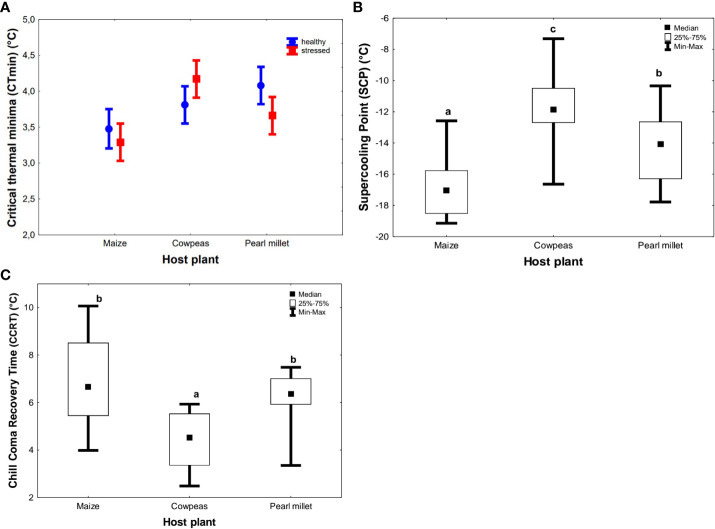
Summary results showing the effects of natural diet (host plant) and treatment on *Spodoptera frugiperda* on cold tolerance **(A)** critical thermal minima (CT_min_), **(B)** supercooling points (SCPs), and **(C)** chill-coma recovery time (CCRT). The circles and boxes in **(A)** represent means for unstressed and stressed plants, respectively, and the error bar represents the standard error of the mean. In boxplots **(B, C)**, the horizontal bars display the median, the box gives the interquartile ranges, and the whiskers show the largest and smallest values up to 1.5 ×  interquartile range. Stressed and unstressed plant results were pooled together for HKDT results since they showed no statistically significant difference. Bars with the same letters are not statistically different from each other at 95% CI (Tukey’s HSD test).

The mean CT_min_ ranged from 3.38°C ± 0.40°C (maize) to 3.87° C ± 0.21°C (pearl millet) and 3.99°C ± 0.19°C (cowpeas) (**Figure 4A**). Unlike for heat-tolerance traits, there were no significant differences in the CT_min_ of FAWs raised on stressed and drought-stressed host plants, and hence the data were pooled. In addition, there was no significant interaction between the type of host plant and drought stress (F_2, 53_ = 1.189, *p* > 0.3124) on CT_min_ (**Table 2**). However, adults that fed on the maize host generally had a higher cold tolerance (lower CT_min_) than all other treatments (**Figure 4A**). SCPs were also generally consistent with CT_min_ results, in which cereal diets (maize and pearl millet) had a higher cold tolerance (more negative SCPs) than more suboptimal cowpeas [Kruskal–Wallis test (H_(2, N = 60)_ = 31.008; *p* < 0.05)]. We recorded the lowest SCPs for maize (−17.05°C ± 0.40°C), which were significantly different from those for pearl millet (−14.06°C ± 0.54°C) and cowpeas (−11.86°C ± 0.47°C) (see **Figure 4B**). Thus, individuals raised on maize had the highest low-temperature tolerance (lowest SCP value), followed by pearl millet and cowpeas (lowest cold tolerance) (**Figure 4B**), and all of these were significantly different from each other. There were significant interactive effects between host plants and their stress condition on both CCRT (*p* = 0.0015) and SCPs (*p* < 0.001). Host plant type had significant effects on CCRT (H_(2, N= 54)_ =18.634 *p* < 0.001) (**Figure 4C**). For CCRT results, the order was the reverse of that for the SCPs results: FAWs adults fed on cowpeas took a significantly shorter time to recover from chill coma (higher cold tolerance) (median of 4.53 minutes ± 0.27 minutes) (*p* < 0.001), followed by those fed on pearl millet (median of 6.37 minutes ± 0.22 minutes) and those fed on maize (highest median of 6.66 minutes ± 0.43 minutes). Maize-fed FAWs were the least cold tolerant when measured as CCRT and were not statistically different from pearl millet. Similar to HKDT and SCP results, drought stress did not have a significant effect on CCRT (H_(1, N= 54)_ = 1.096, *p* > 0.05) (see **Figures 4A–C**).

### 3.4 Relationship between pupal weight and thermal fitness parameters

**Figure 5:**
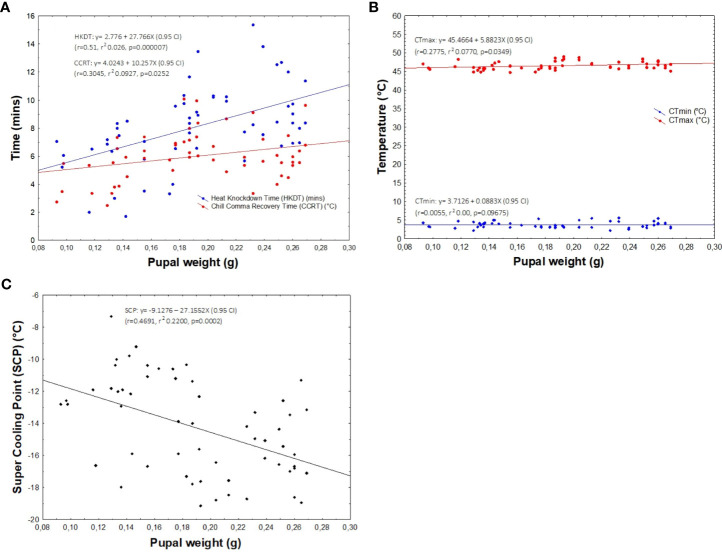
Linear regression analysis assessing the correlation between pupal weight and HKDT and chill-coma recovery time (CCRT) **(A)**, CT_min_ and CT_max_ **(B)**, and SCPs **(C)**.

HKDT was significantly positively correlated to pupal weight (*r* = 0.51, *r*^2^ = 0.026, *p* < 0.05), with heavier pupae having a higher HKDT (or heat tolerance) (**Figure 5A**). A similar trend was observed between pupal weight and CCRT (*r* = 0.305, *r*^2^ = 0.0927, *p* < 0.05), albeit with only a marginal effect (**Figure 5A**). On the other hand, pupal weight had a weak but significant positive correlation with CT_max_ (*r* = 0.278, *r*^2^ = 0.077, *p* = 0.035), whereas no relationship existed between pupal weight and CT_min_ (*r* = 0.006, *r*^2^ = 0.00, *p* = 0.097) (**Figure 5B**), which was consistent with our results showing that there was no significant contribution of the host plant to the observed CT_min_ (see previous section). For SCPs, pupal weight had a moderate significant negative correlation with SCPs (*r* = 0.469, *r*^2^ = 0.2200, *p* < 0.001) (**Figure 5C**).

## 4 Discussion

The FAW is a global herbivorous pest of crops, especially cereals, that has recently spread across divergent geographic spaces (26). It is highly polyphagous and is an opportunistic feeder, with >350 hosts (27). Whereas more optimal hosts, e.g., maize, sorghum, and sugarcane, support their entire development (egg to adult) (26, 50), suboptimal hosts support larval feeding but are rarely reported to support the entire life cycle (21, 22, 26). Our results here show that survival on suboptimal hosts supports the entire life cycle but comes at ecological costs, e.g., of larval size and increased numbers of instars (prolonged developmental period) (26; **Table S1**).

Generally, our results showed that diet quality (water stress) has an effect on FAW life history: directly through body size and growth rates and indirectly through thermal fitness traits. More optimal cereal-based or natural host plant diets [maize and pearl millet (51)] increased FAW larval growth rates, pupal weight, and heat tolerance, with little to no effect on cold tolerance except for SCP. FAWs raised on optimal diets (unstressed maize and pearl millet) grew faster, produced bigger larvae and pupae, and generally had improved heat tolerance compared with those fed suboptimal diets; however, their cold tolerance (except for SCPs) were reduced compared to FAW fed suboptimal diets. Furthermore, there was a direct increase in heat tolerance (HKDT) with increased pupal weight and SCPs, which is indicative of the role of diet in larval size and subsequent stress tolerance. However, cowpeas, a more suboptimal diet (whether stressed or unstressed), slowed down FAW growth rates and reduced heat tolerance compared to those larvae coming from more optimal cereal hosts (maize and pearl millet). Despite feeding on many other host plants (26, 27, 49), faster growth rates on cereal-based hosts support the fact that these two diets optimally support the FAW life cycle (51). Thus, in the multi- or mixed-cropping systems typical of smallholder farmers in sub-Sahara Africa, and when commonly exposed to drought stress, the FAW will (i) complete its full life cycle and (ii) incur fitness costs (for heat tolerance) with unaffected cold-tolerance traits where natural diets are compromised.

Although FAW size (on the basis of larval and pupal weights and developmental rates) and heat-tolerance traits (CT_max_ and HKDT) were compromised when the insect was fed on cowpeas, the result nevertheless showed the species’ resilience, being able to survive and complete its life cycle on cowpeas (suboptimal diets) without significantly altering some of its attributes such as cold tolerance. This compromises the potential use of cowpeas in intercrops as cultural control measures as suggested by other authors (52). The lower larval weights, particularly on stressed cowpeas, however, may be a result of limited feeding due to non-preference and general failure to access the key nutrients required for growth by the larvae. Cowpeas contain low types of carbohydrates and lipids, and have significant quantities of anti-nutritional factors such as oligosaccharides, phytic acid, protease inhibitors, and lectins, as well as polyphenols in the crop biomass (33, 53, 54). The inhibition of protease results in the absence or reduced presence of key amino acids such as tyrosine and phenylalanine, which are key in heat tolerance through the formation of melanin for cuticle hardening (16). The larval FAW developmental stage usually has up to six instars when fed on more optimal diets, but these can vary from 5 to 10 instars when conditions are unfavourable (26, 55, 56). Thus, it appears that FAWs may trade off size and growth rates and increase the number of instars on suboptimal hosts to increase chances of survival (27, 57). Such differences in the number of instars based on the host plant diet and temperature have been well documented in the literature and support our current findings (20, 22, 58, 59). With the current dominance of cowpea production in African small-scale farming systems (28–30), our data show that cowpea (a suboptimal host) may support FAW survival in African farming systems, possibly both in the presence or absence of healthy or unhealthy preferred hosts. Based on our results, FAW populations are unlikely to be significantly affected by the absence of healthy, natural optimal diets in African farming systems.

The successful development, though slow, and retention of high-types cold-tolerance abilities by the FAW when fed on stressed maize and pearl millet shows the strong resilience of the pest and its efficient suboptimal food conversion efficiencies, a key attribute that may aid invasion success in climatically constrained environments. The consistently lower larval weights across all the instars on the stressed than on the unstressed host plants can be attributed to the accumulation of stress-induced metabolites that may act as anti-nutritional factors (60). However, this study did not assess the nutritional composition of the different host plants. Therefore, future studies may aim to quantify the exact nutritional content and quality differences across the host plant conditions to better understand the role of nutrition on ecological and physiological traits. In addition, treatments that had insects that reached eight instars (stressed maize, unstressed and stressed cowpeas, and stressed pearl millet) had low final larval weights compared to those that ended at six instars. This can be attributed to repeated cycles of moulting and high energy demands, which naturally result in weight loss (27, 61), coupled with the limited feeding on those treatments. This is also consistent with the notion that insects reduce their body size and reduce moulting time intervals (more moults and instars) to increase their chances of survival when faced with shocks such as suboptimal diets (18, 22, 62). These findings are similar to those reported by Zhou et al. (22), in which FAW developmental periods were longer on banana (eight larval instars) than maize (six larval instars).

The FAW fed on cowpeas had the highest SCPs (lowest cold tolerance) but had, contrastingly, the shortest CCRT (highest cold tolerance). Traits of the same metric (cold tolerance) may yield different results owing to the differences in underlying mechanisms. For example, CCRT measures the time it takes for insects to regain consciousness following chill coma and recover ion and water homeostasis in muscles upon exposure to more optimal conditions (63). However, SCPs measure the lowest temperature at which an organism can cool before the internal freezing of bodily fluids (48, 64). It is thus not surprising that the cold tolerance traits measured in this study gave varying results. On the other hand, SCP values were significantly lowest for FAW from maize (highest cold tolerance), followed by pearl millet. The type of diet is known to influence SCPs through the concentration of solutes and nucleating agents in the diet that influence the haemolymph content and concentrations (65). We posit that the shorter CCRT could be an intrinsic short-term adaptation by the pest (66) signalled by the exposure to suboptimal diets, signifying harsh conditions that may follow.

Fall armyworms raised on maize and pearl millet recorded higher CT_max_ than those on cowpeas, although both their stressed conditions did not significantly differ from the cowpea-derived individual adult CT_max_. Tolerance to high temperatures is influenced by several factors, which include but are not limited to diet, body size, physiological responses, and evolutionary history (25, 67–70). This is likely to be a direct result of investment in carbohydrates from the crops as well as the larger body sizes, which help in buffering against the effects of high temperatures (67) (25). Our results concur with the previous findings by Nyamukondiwa and Terblanche (69) that showed that thermal tolerance may be an active process and that requires high energy reserves, and therefore more optimal, carbohydrate-rich diets, support higher thermal fitness. Furthermore, the results also concur with Segaiso et al. (39), who found that fasting causes significant thermal fitness costs in FAW larvae. Although insect size is known to positively correlate with heat tolerance, it is not always the case with all species, particularly with some small temperate insects, e.g., North American ants (5). Our findings also showed that stressed natural hosts confer thermal fitness costs on FAW adults, possibly due to limited feeding and failure to acquire key nutrients (as in 39).

Our findings show that exposure of FAWs to suboptimal diets (stressed or non-preferred host plants) will confer thermal fitness costs on the insects, particularly in the context of increasing temperatures or overwintering potential (high supercooling ability). However, populations raised on suboptimal hosts may recover faster when subjected to acute low temperatures, and hence have high chances of survival. On the other hand, the ability of FAWs to complete their development on cowpeas (both unstressed and stressed) threatens the efficacy of intercropping or crop rotation as a management strategy for this pest in integrated pest management programmes, particularly in small-scale farming systems.

The significant positive correlation between both CT_max_ and HKDT and pupal weight showed that insect size, as influenced by different diets, significantly affected heat tolerance, with heavier/bigger pupa having better tolerance to heat than small ones. This is possibly because of the increased production of heat shock proteins and increased thermal inertia (71). On the other hand, the moderate relationship between pupal weight and SCPs showed that insect diet had an effect on cold tolerance, possibly through the concentration of adequate solutes from the optimal diets.

## 5 Conclusions

Fall armyworm physical development and thermal fitness were significantly influenced by diet. Although the non-preferred and drought-stressed host plants reduced heat tolerance and increased the number of larval instars, they did not impair full development. Effects on cold tolerance were equivocal, but SCPs were negatively affected by suboptimal diets. We thus conclude that (1) FAWs faced with suboptimal diets in wild habitats will have prolonged developmental time (more instars), reduced fitness (low larval and pupal weights), reduced growth rates, and reduced physiological fitness, which has cascading effects on abiotic stress tolerance; and (2) the FAW has dietary resilience and the pest can survive on suboptimal diets (non-preferred or drought-stressed plants) typical in rain-fed, multi-cropping ecosystems common to most rural farming systems in Africa. These results suggest host plant effects on ecological and physiological performance of the FAW and thus the fate of population persistence for this pest under multivariate environments may be complex and require consideration of prior environmental host plant history. Use of suboptimal diets by insect pests in crop rotations and/or mixtures may thus have far-reaching consequences as both costs and gains and may derail benefits of multi-cropping systems, as used in integrated crop- and pest-management strategies.

Although FAW managed to develop on stressed plants, there is a need to investigate whether or not they are able to develop in a random host-switching scenario or extremely drought-stressed natural diets as they grow. Thus, further work is needed to see the interaction effects of multiple stressed or unstressed host switching during the larval stages on FAW survival. Such within-generation host switching can be a key requisite for the survival of invasive species under multivariate food-limited environments. Further studies on insect hormonal changes and the role of adaptive physiological mechanisms such as heat-shock proteins on insects fed on suboptimal diets is warranted to further explain the observed trends. There is also a need to establish the link between host plant nutritional composition (including available phytochemical compounds) and the role of gut microbes in metabolising recalcitrant or suboptimal food diets. Further studies are required to investigate the biological characteristics (oviposition, fecundity, and survival capacity) of FAWs when exposed to different durations of stress (continuous or intermittent) and thermal responses of the subsequent generations.

## Data availability statement

The original contributions presented in the study are included in the article/**Supplementary Material**. Further inquiries can be directed to the corresponding author.

## Ethics statement

The manuscript presents research on animals that do not require ethical approval for their study.

## Author contributions

Project conceptualisation and management: MM, HM, and CN. Data curation: MM and BS. Formal analysis: MM, HM, and CN. Investigation: MM and HM. Visualisation and validation: MM, HM, FC, BM, BS, and CN. Writing—original draft: MM, HM, and CN. Writing—review and editing: MM, HM, FC, BM, BS, and CN. Funding: CN and HM. All authors contributed to the article and approved the submitted version.
