# Supplementary material for: Sub-optimal host plants have developmental and thermal fitness costs to the invasive fall armyworm
Source: Front Insect Sci. 2023 Sep 29;3:1204278. doi: 10.3389/finsc.2023.1204278 (PMC10926449; doi:10.3389/finsc.2023.1204278)
Supplement: Supplementary file 1 [file DataSheet_1.docx]

**Sub-optimal host plants have developmental and thermal fitness costs to the invasive fall armyworm**

**Macdonald Mubayiwa^1^; Honest Machekano^2^*; Frank Chidawanyika^3,4^; Brighton M. Mvumi^5^, Bame Segaiso^1^ and Casper Nyamukondiwa^1,6^**

^1^Department of Biological Sciences and Biotechnology, Botswana International University of Science and Technology, Palapye, Botswana

^2^Department of Zoology and Entomology, University of Pretoria, Pretoria, South Africa

^3^International Centre of Insect Physiology and Ecology (ICIPE), Plant Health Department, Nairobi, Kenya

^4^Department of Zoology and Entomology, University of the Free State, Bloemfontein, South Africa

^5^ Department of Agricultural and Biosystems Engineering, Faculty of Agriculture, Environment and Food Systems, University of Zimbabwe, Harare, Zimbabwe

^6^Department of Zoology and Entomology, Rhodes University, Makhanda, 6140, South Africa

***Correspondence:**

Corresponding author: [honest.machekano@up.ac.za](mailto:honest.machekano@up.ac.za)

**Table S1:** Summary of how different host plants and/or diets affect the FAW growth and development as reflected by number of larval instars and duration of the larval developmental stage

| **Host plant/diet** | **Rearing temperature (°C)** | **Number of larval instars** | **Duration of larval stage (days)** | **References** |
| --- | --- | --- | --- | --- |
| Unstressed maize |  | 6 | - | (He et al., 2021a; Zhou et al., 2022), current study |
| Water-stressed maize |  | 8 | - | current study |
| Unstressed cowpeas |  | 8 | - | current study |
| Water-stressed cowpeas |  | 8 | - | current study |
| Unstressed pearl millet |  | 6 | - | current study |
|  | 25 | 4 | 25.153 | (Carvalho *et al.*, 2022) |
| Water-stressed pearl millet |  | 8 | - | current study |
| Banana | 25 | 9 | 67.32 | (Zhou *et al.*, 2022) |
| Maize | 19 | - | 39.21*^[[1]](#footnote-1)^ | (Huang *et al.*, 2021) |
|  | 23.9 | - | 17.4 | (Meagher and Nagoshi, 2012) |
|  | 20 | 7 | 24.16 | (Chen *et al.*, 2022) |
|  | 22 | - | 21.70* | (Huang *et al.*, 2021) |
|  | 25 | 7 | 14.87 | (Chen *et al.*, 2022) |
|  |  | 6 | 20.51 | (Zhou *et al.*, 2022) |
|  | 28 | - | 12.94* | (Huang *et al.*, 2021) |
|  | 30 | 7 | 9.3 | (Chen *et al.*, 2022) |
|  | 31 | - | 11.05* | (Huang *et al.*, 2021) |
| Star grass | 23.9 | - | 23.0 | (Meagher and Nagoshi, 2012) |
| Pinto bean | 23.9 | - | 19.3 | (Meagher and Nagoshi, 2012) |
| Cotton | 25 | - | 22 | (Pitre & Hogg, 1983) |
|  | 25-30 | 7 | 24.2 | (Wang *et al.*, 2020) |
| Soybean | 25 | 7 | 23.3 | (He et al., 2021b) |
|  | 26 | - | 5.5 | (Boregas *et al.*, 2013) |
| Cow peas | 23.9 | - | 23.3 | (Meagher et al., 2004) |
| Sunn hemp | 23.9 | - | 23.4 | (Meagher et al., 2004) |
|  | 28 | 7 | 19.82 (F)  21.28 (M) | (Ashok et al., 2020) |
| Sorghum-Sudangrass | 23.9 | - | 19.7 | (Meagher et al., 2004) |
| Sugar cane | 25 |  | 16.82 | (Kranthi and Devi, 2021) |
|  | 26 | - | 7.4 | (Boregas *et al.*, 2013) |
| Artificial diet | 25 |  | 12.28 | (Kranthi and Devi, 2021) |
| Rice | 25 | 6 | 32.74 | (Altaf *et al.*, 2022) |
|  | 26 | - | 6.7 | (Boregas *et al.*, 2013) |
| Wheat | 25 | 6 | 22.2 | (Altaf *et al.*, 2022) |
|  | 25 | 7 | 17.9 | (Gebretsadik *et al.*, 2023) |
| Sorghum | 20 | 7 | 24.1 | (Chen *et al.*, 2022) |
|  | 25 | 6 | 19.2 | (Altaf *et al.*, 2022) |
|  |  | 7 | 17.39 | (Chen *et al.*, 2022) |
|  | 30 | 7 | 10.68 | (Chen *et al.*, 2022) |
| Napier grass (*Pennisetum purpureum*) | 28 | 7 | 18.73* | (Chen *et al.*, 2023) |
| Natal grass (*Melinis repens*) | 28 | 8 | 20.92* | (Chen *et al.*, 2023) |
| Coix seed (*Coix lacryma-jobi* L.)) | 20 | 7 | 24.08 | (Chen *et al.*, 2022) |
|  | 25 | 7 | 18.33 | (Chen *et al.*, 2022) |
|  | 30 | 7 | 11.45 | (Chen *et al.*, 2022) |
| Pepper (*Capsicum annuum* L.) | 25 | 7 | 29.41 | (Wu *et al.*, 2021) |
| Tomato (*Solanum lycopersicum* Mill.) | 25 | 7 | 23.5 | (Wu *et al.*, 2021) |
|  | 25-30 | 7 | 26.28 | (Wang *et al.*, 2020) |
| Eggplant (*Solanum melongena* L.) | 25 | 4 | 20.12 | (Wu *et al.*, 2021) |
| Sunflower | 25 | 7 | 17.6 | (He et al., 2021b) |
| Oil seed rape | 25 | 6 | 21.2 | (He et al., 2021b) |
| Faba beans | 25 | 7 | 20.99 | (Gebretsadik *et al.*, 2023) |
| Soya beans | 25 | 7 | 17.61 | (Gebretsadik *et al.*, 2023) |
| Barley | 25 | 7 | 20.13 | (Gebretsadik *et al.*, 2023) |
| Chinese cabbage | 25-30 | 7 | 30.66 | (Wang *et al.*, 2020) |
| Sourgrass | 26 | - | 25 | (Moraes *et al.*, 2020) |
| Johnsongrass | 26 | - | 15.5 | (Moraes *et al.*, 2020) |
| Goosegrass | 26 | - | 18.3 | (Moraes *et al.*, 2020) |
| Pork caruru | 26 | - | 22.5 | (Boregas *et al.*, 2013) |
| Peanut | 26 | - | 17.7 | (Boregas *et al.*, 2013) |
| Maranda grass | 26 | - | 14.7 | (Boregas *et al.*, 2013) |
| Signal grass | 26 | - | 16.6 | (Boregas *et al.*, 2013) |
| Butter grass | 26 | - | 20.3 | (Boregas *et al.*, 2013) |
| Potato grass | 26 | - | 27.1 | (Boregas *et al.*, 2013) |
| Bean | 26 | - | 12.9 | (Boregas *et al.*, 2013) |
| Wild sorghum | 26 | - | 18.1 | (Boregas *et al.*, 2013) |
| Soybean flour and wheat bran artificial diet | 25 | - | 14.1 | (He et al., 2021b) |
| Maize leaf and common leaf powder diet | 27 | 6 | 13.95 | (Ashok *et al.*, 2021) |
| Common bean powder diet | 27 | 6 | 17.32 | (Ashok *et al.*, 2021) |
| Maize leaf and chickpea flour diet | 27 | 6 | 19.82 | (Ashok *et al.*, 2021) |
| Maize leaf powder and maize flour diet | 27 | 6 | 22.46 | (Ashok *et al.*, 2021) |
| Rice leaf powder and chickpea flour diet | 27 | 6 | 11.20 | (Ashok *et al.*, 2021) |
| Chickpea flour diet | 27 | 6 | 20.05 | (Ashok *et al.*, 2021) |
| Bean diet | 25 | - | 15.6 | (Pinto *et al.*, 2019) |
| Corn flour diet | 25 | - | 34.5 | (Pinto *et al.*, 2019) |
| Green corn diet | 25 | - | 5.3 | (Pinto *et al.*, 2019) |
| Sweetcorn kernels | 18 | 6 | 34.9 | (Du Plessis et al., 2020) |
|  | 22 | 6 | 20.58 | (Du Plessis et al., 2020) |
|  | 26 | 6 | 14.86 | (Du Plessis et al., 2020) |
|  | 30 | 6 | 11.38 | (Du Plessis et al., 2020) |
|  | 32 | 6 | 10.45 | (Du Plessis et al., 2020) |

Table S2: Overall mean interactive effects of host and stress status on FAW growth rates, larval and pupal weight (n = 10)

| **Treatment** | **Larval weight (6th instar)** | **Daily larval weight gain** | **Pupal weight** |
| --- | --- | --- | --- |
| Unstressed maize | 0.8 ± 0.02 | 0.05 ± 0.00e | 0.23 ± 0.01 |
| Stressed maize | 0.7 ± 0.03 | 0.04 ± 0.00c | 0.20 ± 0.01 |
| Unstressed cowpeas | 0.58 ± 0.02 | 0.02 ± 0.00ab | 0.14 ± 0.01 |
| Stressed cowpeas | 0.48 ± 0.03 | 0.02 ± 0.00a | 0.12 ± 0.01 |
| Unstressed pearl millet | 0.73 ± 0.04 | 0.04 ± 0.00d | 0.21 ± 0.01 |
| Stressed pearl millet | 0.65 ± 0.03 | 0.03 ± 0.00b | 0.17 ± 0.01 |
| p-value | 0.907 | 0.014 | 0.59 |
| F_5, 54_ | 0.10 | 4.62 | 0.53 |

**Table S3.** Host plant and stress condition interaction on thermal fitness parameters of fall armyworm adults (n = 10)

| **Treatment** | **CT max (°C)** | **HKDT (min)** | **CT min (°C)** | **CCRT (min)** | **SCP (°C)** |
| --- | --- | --- | --- | --- | --- |
| Unstressed maize | 47.71 ± 0.29c | 8.44 ± 0.37c | 3.48± 0.02 | 6.45 ± 0.56bc | -16.88±0.67a |
| Stressed maize | 46.56 ± 0.26ab | 8.84 ± 0.63c | 3.29± 0.03 | 7.37 ± 0.64c | -16.93±0.47a |
| Unstressed cowpeas | 45.82 ± 0.28a | 3.37 ± 0.46a | 3.81± 0.02 | 5.33 ± 0.22b | -10.92±0.35b |
| Stressed cowpeas | 46.24 ± 0.35ab | 6.89 ± 0.30b | 4.17± 0.04 | 3.40 ± 0.21a | -12.44±0.82b |
| Unstressed pearl millet | 47.03 ± 0.36bc | 11.80 ± 0.68d | 4.08± 0.04 | 5.60 ± 0.31b | -16.13±0.46a |
| Stressed pearl millet | 46.02 ± 0.14a | 7.87 ± 0.43bc | 3.66± 0.02 | 6.88 ± 0.16c | -12.34±0.45b |
| p-value | 0.018 | <.001 | 0.312 | 0.002 | <.001 |
| F_5, 54_ | 4.3 | 24.08 | 1.189 | 7.446 | 11.98 |

*Means with the same letter within a column are not significantly different from each other based on Fishers Protected LSD test at 95 % confidence interval.*

**Fig S1.** Overall treatment effects on pupal weights showing significant effect of drought stress on pupal weight of *S. frugiperda*, regardless of the natural diet (host plant). Comparison based on Tukey’s HSD test, F _(1, 53)_ = 6.3719, p = 0.01462.

**References**

Altaf, N., Idrees, A., Ullah, M. I., Arshad, M., Afzal, A., Afzal, M., Rizwan, M., & Li, J. (2022). Biotic Potential Induced by Different Host Plants in the Fall Armyworm, *Spodoptera frugiperda* (Lepidoptera: Noctuidae). *Insects*, *13*(10), 1–10. https://doi.org/10.3390/insects13100921

Ashok, K., Balasubramani, V., Kennedy, J. S., Geethalakshmi, V., Jeyakumar, P., & Sathiah, N. (2021). Evaluating Artificial Diets for the Fall Armyworm, *Spodoptera frugiperda* (J.E. Smith) (Lepidoptera: Noctuidae) through Nutritional Indices and an Age-Stage, Two-Sex Life Table Approach. *African Entomology*, *29*(2), 620–634. https://doi.org/10.4001/003.029.0620

Boregas, K. G. B., Mendes, S. M., Waquil, J. M., & Wilson Fernandes, G. (2013). Fitness stage of *Spodoptera frugiperda* (J. E. Smith) (Lepidoptera: Noctuidae) on alternative hosts. *Bragantia*, *72*(1), 61–70. https://doi.org/10.1590/S0006-87052013000100009

Carvalho, I. F., Machado, L. L., Neitzke, C. G., Erdmann, L. L., Oliveira, L. T., Bernardi, D., & Rosa, A. P. S. A. da. (2022). Biological Parameters and Fertility Life Table of *Spodoptera frugiperda* in Different Host Plants. *Journal of Agricultural Science*, *14*(10), 48. https://doi.org/10.5539/jas.v14n10p48

Chen, W., Itza, B., Kafle, L., & Chang, T. (2023). Life Table Study of Fall Armyworm *(Spodoptera frugiperda)* (Lepidoptera: Noctuidae) on Three Host Plants under Laboratory Conditions. *Insects*, *14*, 329.

Chen, Y. C., Chen, D. F., Yang, M. F., & Liu, J. F. (2022). The Effect of Temperatures and Hosts on the Life Cycle of *Spodoptera frugiperda* (Lepidoptera: Noctuidae). *Insects*, *13*(2), 1–15. https://doi.org/10.3390/insects13020211

Du Plessis, H., Schlemmer, M., & Van den Berg, J. (2020). The Effect of Temperature on the Development of *Spodoptera fruigiperda* (Lepidoptera: Noctuidae). *Insects*, *11*(228), 1–11.

Gebretsadik, K. G., Liu, Y., Yin, Y., Zhao, X., & Li, X. (2023). Population growth of fall armyworm, *Spodoptera frugiperda* fed on cereal and pulse host plants cultivated in Yunnan Province, China. *Plants*, *12*, 950.

He, L, Wang, T., Chen, Y., Ge, S., Wychuys, K. A. ., & Wu, K. (2021). Larval diet affects development and reproduction of East Asian strain of the fall armyworm, *Spodoptera frugiperda*. *Journal of Integrative Agriculture*, *20*(3), 736–744. https://doi.org/10.1016/S2095-3119(19)62879-0

He, Li mei, Wu, Q. lin, Gao, X. wu, & Wu, K. ming. (2021). Population life tables for the invasive fall armyworm, *Spodoptera frugiperda* fed on major oil crops planted in China. *Journal of Integrative Agriculture*, *20*(3), 745–754. https://doi.org/10.1016/S2095-3119(20)63274-9

Huang, L. L., Xue, F. Sen, Chen, C., Guo, X., Tang, J. J., Zhong, L., & He, H. M. (2021). Effects of temperature on life-history traits of the newly invasive fall armyworm, *Spodoptera frugiperda* in Southeast China. *Ecology and Evolution*, *11*(10), 5255–5264. https://doi.org/10.1002/ece3.7413

Kranthi, P., & Devi, R. S. (2021). Comparative Biology of Fall Armyworm, *Spodoptera frugiperda* on Different Host Plants under Laboratory Conditions. *Biological Forum - An International Journal*, *13*(4), 381–387.

Meagher, R.L., Nagoshi, R.N., Stuhl, C., Mitchell, E.R. (2004) Larval development of fall armyworm (Lepidoptera: Noctuidae) on different cover crop plants. *Florida Entomologist*, 87, 454–60

Meagher, R. L., & Nagoshi, R. N. (2012). Differential feeding of fall armyworm (Lepidoptera: Noctuidae) host strains on meridic and natural diets. *Annals of the Entomological Society of America*, *105*(3), 462–470. https://doi.org/10.1603/AN11158

Moraes, T., Ferreira Da Silva, A., Leite, N. A., Karam, D., & Mendes, S. M. (2020). Survival and Development of Fall Armyworm (Lepidoptera: Noctuidae) in Weeds during the Off-Season. *Florida Entomologist*, *103*(2), 288–292. https://doi.org/10.1653/024.103.0221

Pinto, J. R. L., Torres, A. F., Truzi, C. C., Vieira, N. F., Vacari, A. M., & De Bortoli, S. A. (2019). Artificial Corn-Based Diet for Rearing *Spodoptera frugiperda* (Lepidoptera: Noctuidae). *Journal of Insect Science*, *19*(4), 1–8. https://doi.org/10.1093/jisesa/iez052.

Pitre, H.N., Hogg, D.B. (1983). Development of the fall armyworm on cotton, soybean and corn. *Journal of the Georgia Entomological Society,* 18, 187-194

Wang, W., He, P., Zhang, Y., Liu, T., Jing, X., & Zhang, S. (2020). The population growth of *Spodoptera frugiperda* on six cash crop species and implications for its occurrence and damage potential in China. *Insects*, *11*(9), 1–14. https://doi.org/10.3390/insects11090639

Wu, L. hong, Zhou, C., Long, G. yun, Yang, X. bin, Wei, Z. yan, Liao, Y. jiang, Yang, H., & Hu, C. Xing. (2021). Fitness of fall armyworm, *Spodoptera frugiperda* to three solanaceous vegetables. *Journal of Integrative Agriculture*, *20*(3), 755–763. https://doi.org/10.1016/S2095-3119(20)63476-1

Zhou, S., Qin, Y., Wang, X., Zheng, X., & Lu, W. (2022). Fitness of the fall armyworm Spodoptera frugiperda to a new host plant, banana (Musa nana Lour.). *Chemical and Biological Technologies in Agriculture*, *9*(78), 1–9. https://doi.org/10.1186/s40538-022-00341-z

1. Weighted average of developmental time of male and female larvae. [↑](#footnote-ref-1)
